# SRRM2, a Potential Blood Biomarker Revealing High Alternative Splicing in Parkinson's Disease

**DOI:** 10.1371/journal.pone.0009104

**Published:** 2010-02-08

**Authors:** Lina A. Shehadeh, Kristine Yu, Liyong Wang, Alexandra Guevara, Carlos Singer, Jeffery Vance, Spyridon Papapetropoulos

**Affiliations:** 1 Department of Molecular and Cellular Pharmacology, University of Miami Leonard M. Miller School of Medicine, Miami, Florida, United States of America; 2 Department of Human Genetics, University of Miami Leonard M. Miller School of Medicine, Miami, Florida, United States of America; 3 Department of Neurology, University of Miami Leonard M. Miller School of Medicine, Miami, Florida, United States of America; National Institutes of Health, United States of America

## Abstract

**Background:**

Parkinson's disease (PD) is a progressive neurodegenerative disorder that affects about five million people worldwide. Diagnosis remains clinical, based on phenotypic patterns. The discovery of laboratory markers that will enhance diagnostic accuracy, allow pre-clinical detection and tracking of disease progression is critically needed. These biomarkers may include transcripts with different isoforms.

**Methodology/Principal Findings:**

We performed extensive analysis on 3 PD microarray experiments available through GEO and found that the RNA splicing gene **SRRM2** (or SRm300), sereine/arginine repetitive matrix 2, was the only gene differentially upregulated among all the three PD experiments. SRRM2 expression was not changed in the blood of other neurological diseased patients versus the healthy controls. Using real-time PCR, we report that the shorter transcript of SRRM2 was 1.7 fold (*p = 0.008*) upregulated in the substantia nigra of PDs vs controls while the longer transcript was 0.4 downregulated in both the substantia nigra (*p = 0.03*) and amygdala (*p = 0.003*). To validate our results and test for the possibility of alternative splicing in PD, we performed independent microarray scans, using Affymetrix Exon_ST1 arrays, from peripheral blood of 28 individuals (17 PDs and 11 Ctrls) and found a significant upregulation of the upstream (5′) exons of SRRM2 and a downregulation of the downstream exons, causing a total of 0.7 fold down regulation (*p = 0.04*) of the long isoform. In addition, we report novel information about hundreds of genes with significant alternative splicing (differential exonic expression) in PD blood versus controls.

**Conclusions/Significance:**

The consistent dysregulation of the RNA splicing factor SRRM2 in two different PD neuronal sources and in PD blood but not in blood of other neurologically diseased patients makes SRRM2 a strong candidate gene for PD and draws attention to the role of RNA splicing in the disease.

## Introduction

The diagnosis of Parkinson's disease (PD) remains purely clinical and is based on the phenotypic expression of three cardinal clinical signs (bradykinesia, tremor and rigidity) in the absence of symptoms and signs indicative of other neurological diseases. Genome-wide transcription analysis of biological material (i.e. human, animal tissues, cell cultures) has shown promise in identifying disease-relevant biomarkers [1]. Postmortem human brain expression profiling has shown significant changes in PD versus healthy states [2], [3], [4], [5], [6], [7], [8], [9], [10]. A single study looking at expression patterns in peripheral venous blood samples of patients with movement disorders and control subjects has provided some preliminary insight on possible blood biomarker candidates for PD diagnosis [11].

We hypothesized that computational analysis of multiple PD microarray data can be used to identify certain sets of PD-associated marker genes based on gene expression patterns. Therefore, we thoroughly analyzed three publicly available PD datasets generated from a rotenone-treated neuroblastoma line[12], postmortem substantia nigra from PD patients and controls[2], and lymphocytes from PD patients and controls[11]. While this approach deals with a huge variability factor, the strength of the approach lies in using evidence in multiple PD datasets that may be very different in etiology, cause, and tissue source. In summary, we hypothesized that interrogating publicly available PD transcriptome-wide expression datasets from different tissue sources would identify lead candidate genes.

In this study, we identified the RNA splicing factor **SRRM2** (or SRm300), serine/arginine (SR) repetitive matrix 2 as differentially expressed in multiple gene expression datasets. Such an SR protein binds both to sequence elements on the pre-mRNA and to other components of the spliceosome. As a result, multimolecular complexes that interact with pre-mRNA at multiple sites are formed, allowing the recognition and bringing together of distant sequences in the excision-splicing event[13]. Driven by the biological function of SRRM2, we investigated the possibility of alternative splicing of 1) this candidate gene in two different brain regions from PD patients and controls, and 2) global transcripts in PD blood samples and controls. We found that SRRM2 has differential splicing in PD substantia nigra, amygdala, and leukocytes of sporadic PD patients. In addition to identifying SRRM2 as alternatively spliced and as a potential biomarker for PD, we report here 3 general interesting findings. First, there is an interesting overlap in differential gene expression between the brain (substantia nigra) and blood in PD, which highlights the credibility for using blood to find biomarkers in PD. Second, there are 35 genes (including SRRM2) differentially expressed in PD blood in both our new blood study and the Scherzer blood study[11]. These may all be worth further scrutiny as potential blood biomarkers for PD. Third, there are hundreds of genes (including SRRM2) with differential alternative splicing in PD blood samples relative to controls. Therefore, there is a prevalent phenomenon of alternative splicing in PD that calls for more study and investigation.

## Results

### SRRM2 Is the Only Differentially Expressed Gene in All 3 PD Expression Datasets

**Figure 1:**
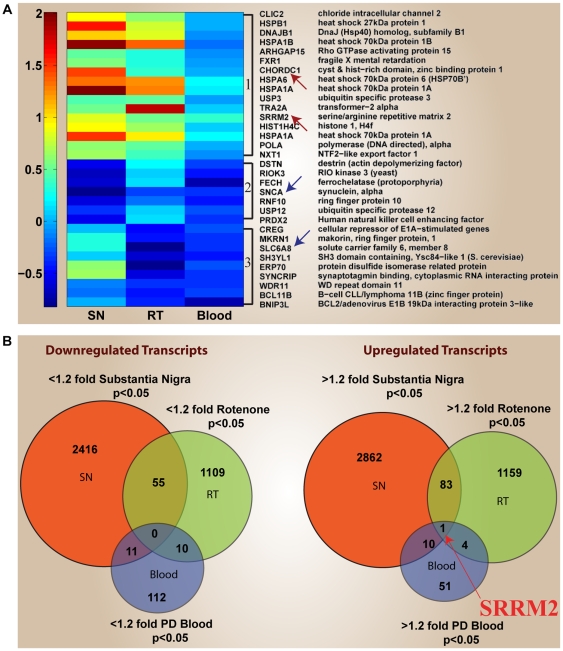
Heat map and venn diagrams of differentially expressed genes overlapping in multiple PD studies. **A.** Shown are the fold change expression levels of genes differentially expressed in at least 2/3 PD studies: (1) up in both the substantia nigra (SN) and 4-wk rotenone-treated cells (RT), (2) down in substantia nigra and blood, (3) down in 4-wk rotenone-treated cells and blood. Arrows point to selected upregulated (red) and downregulated (blue) genes. **B.** Differentially expressed genes determined by our analysis of 3 different microarray experiments on PD, each from a different tissue source, were compared to find common downregulated (left) and upregulated (right) transcripts. A 1.2 fold change and *p<.05* significance cut-offs were used. No multiple correction was employed.

We performed extensive analysis on three different publicly available microarray experiments on PD: (A) Twenty-two chips from the substantia nigra in postmortem brain of PD patients and controls [2], (B) 21 chips on one to four week rotenone-treated neuroblastoma cells [12] (an *in vitro* model of PD), (C) 105 chips on peripheral blood of PD versus healthy and neurological disease control subjects [11]. There were 174 transcripts corresponding to 160 genes that overlapped in at least 2/3 PD experiments. Representative genes are shown in the heatmap (Figure 1A). The complete list of differentially expressed overlapping genes between any 2 of the 3 PD experiments is presented in Table S1. The RNA splicing gene SRRM2, (serine/arginine repetitive matrix 2) was the only gene differentially upregulated in all three PD experiments (Figure 1B). SRRM2 transcript was 1.77 fold (*p<0.01*) upregulated in the substantia nigra of PD patients versus controls. SRRM2 transcript was 1.44 fold (*p<0.05*) upregulated in the 4 wk rotenone treated cells versus the controls. SRRM2 transcript was 1.27 fold (*p<0.05*) upregulated in peripheral blood of PD patients versus healthy controls and was unchanged in the blood of neurological disease patients versus the healthy controls.

### SRRM2 Isoforms Are Both Differentially Expressed in PD but in Opposite Directions

**Figure 2:**
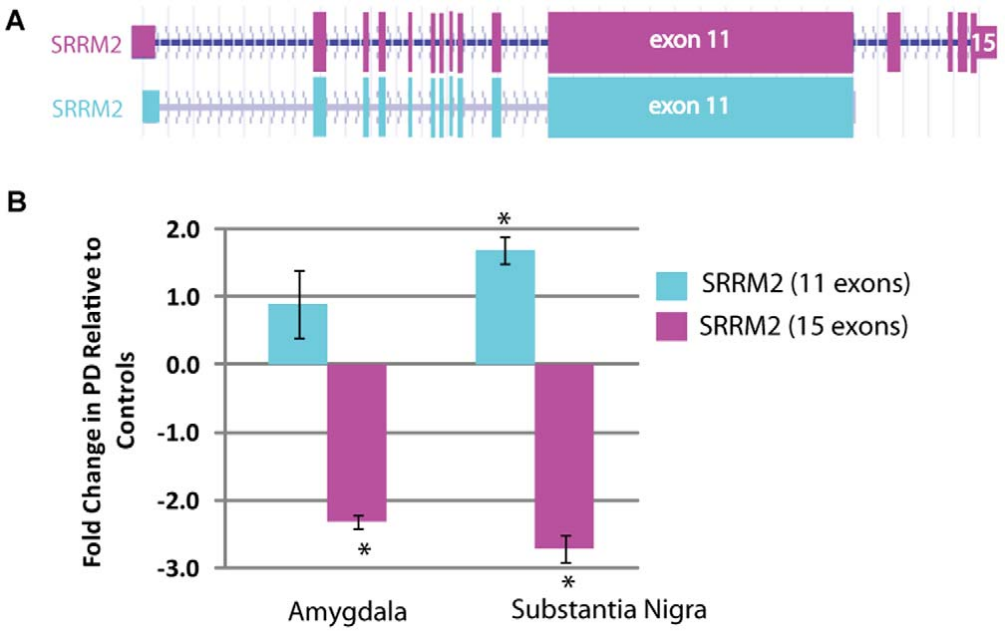
Alternate isoforms of SRRM2 with different number of exons and different expression levels in postmortem PD brain. **A.** Two main splice variants of SRRM2 differ at their 3′ end. The longer SRRM2 isoform contains 15 exons and the shorter isoform contains 11 exons. **B.** SRRM2 isoforms were differentially expressed in postmortem PD brain regions. The shorter transcript of SRRM2 was 1.7 fold (*p = 0.008*) upregulated in the SN of PDs versus controls while the longer transcript was 0.4 fold downregulated in both the SN (*p = 0.03*) and Amygdala (*p = 0.003*) of PDs versus controls.

SRRM2 has one long isoform (16 exons) and 3 short isoforms (∼11 exons). See Figure 2A. Using real-time PCR on postmortem substantia nigra (SN) and amygdala from 10 PD patients versus 10 controls, we found that the shorter transcript of SRRM2 was significantly upregulated (FC = 1.7, *p = 0.008*) in the SN (as in the microarrays) but unchanged in the amygdala (AMG) of PD patients. Interestingly, the longer transcript of SRRM2 was significantly 0.4 downregulated in both the SN (*p = 0.03*) and Amygdala (*p = 0.003*) of PD patients (Figure 2B).

### Splicing Analysis Confirms Isoform Switching of SRRM2 and Reveals High Alternative Splicing Phenomenon in PD

**Figure 3:**
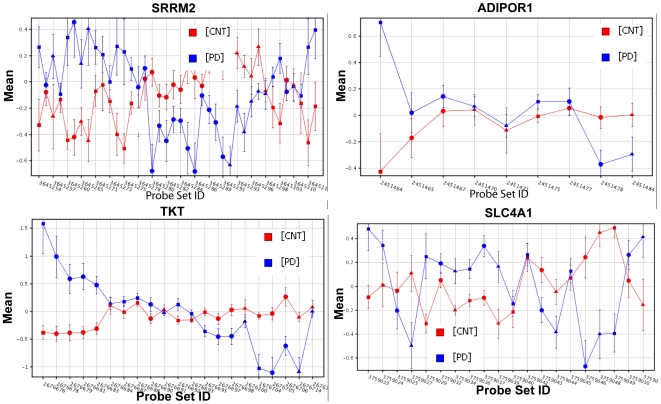
Differential Exonic expression in PD Blood. Splicing Analysis reveals significant differential exonic expression within 218 genes (*p<0.05* with Benjamini Hochberg FDR correction). Shown here are SRRM2, ADIPOR1, TKT, and SLC4A1, all four genes were also differentially expressed in the Scherzer blood data (*p<0.05* with no multiple correction). Each probeset represents an exon. Note that in SRRM2, the 5′ exons are upregulated while the downstream exons are downregulated in PD patients versus controls.

**Figure 4:**
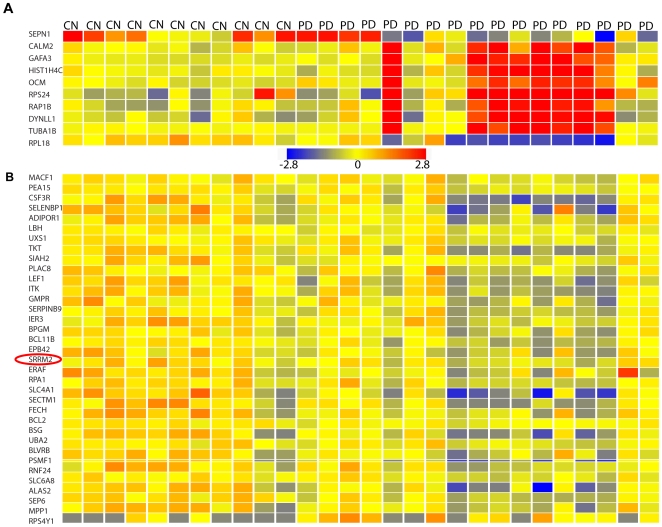
Differential gene expression in 2 PD blood studies. **A.** Shown are top 10 genes differentially expressed by at least 2.5 fold in PD blood versus controls according to analysis of our new expression arrays (*p<0.05* with no multiple correction). **B.** 35 differentially expressed transcripts by at least 1.2 fold overlap between our new PD blood profiling and the Scherzer blood dataset (*p<0.05* with no multiple correction).

Using new Exon array profiling on peripheral blood that we collected from 17 PD patients and 11 controls (GSE 18838), we looked for genes with differential splicing. We identified exons that have statistically significant changes in inclusion rates (relative to the gene level) between PDs and controls. Consistent with rt-PCR results, we found that SRRM2 in PD switches from basal transcript levels of SRRM2 to high expression of the short isoform and low expression of the long isoform. Interestingly, there was a strikingly significant elevation of overall gene splicing in PD blood compared to controls as shown by 218 genes (listed in Table S2), each consisting of exons that have differential expression within the same gene between PDs and controls. Figure 3 shows the differential exonic expression of SRRM2 and 3 other genes. These 4 genes are the ones that overlapped between the 218 differentially spliced genes in our new dataset and the 35 genes that we validated from the Scherzer blood microarray [11] (as explained in the next section and shown in Figure 4B). Gene Ontology classification of the 218 genes with significant splicing events grouped 112 of the 218 genes into one molecular significant function, protein binding, with corrected *p-value  = 0.004*, (listed in Table S3).

### Comparison with Earlier Blood Expression Study

Our new microarray experiment examining global gene expression in peripheral blood samples from 17 PD patients and 11 controls revealed 58 genes differentially expressed by at least 2.0 fold change (*p<0.05*) (listed in Table S4). The top 10 genes (FC of at least 2.5) are shown in Figure 4A. We compared the genes differentially expressed (FC = 1.2 *p<0.05*) in our data to the ones in the Scherzer data [11]. Interestingly, we found an overlap of 35 transcripts shown in Figure 4B, including the downregulated Bcl11B which had previously been validated by real-time PCR [11]. That is out of ∼150 transcripts that were differentially expressed in either one of the two PD blood expression studies (∼40,000 transcripts queried per chip), 35 genes, including SRRM2, were common to both studies. Note that in our microarray data, not all genes including SRRM2 passed the threshold when we stratified by gender as shown in Supplementary Figure S1. This is most likely due to loss of power as the number of replicates became small after stratification.

## Discussion

We interrogated multiple datasets from different tissue sources and identified a potential molecular marker SRRM2 (Figure 1B). While SRRM2 was the only gene that stood out as differentially expressed in all 3 PD public datasets, there were other genes that overlapped in 2 out of the 3 PD experiments (Figure 1A and Table S1). Among the intriguing overlaps that emerged from our analysis are the following: SNCA (alpha-synuclein), a PD-associated gene involved in microglial cell activation and synaptic transmission, was significantly downregulated in both the substantia nigra and blood in PD patients versus controls. BCL11B, which was previously shown by microarrays and rt-PCR to be downregulated in PD blood[11] was also downregulated in rotenone-treated neuroblastomas, and so was the creatine neurotransmitter transporter SLC6A8. The transcription factor SOX11 and the endoplasmic reticular protein OLFM1, both involved in nervous system development, as well as CNTN4 (contactin4) involved in axonal guidance and brain development, were all downregulated in both the substantia nigra and rotenone-treated neuroblastomas.

SRRM2 plays an important role in pre-mRNA splicing and has recently been shown to play a role in cell migration [14]. Recent analyses suggest that at least 75% of multi-exon genes in the human genome are subject to alternative splicing [15]. This form of pre-mRNA post-transcriptional modification has the potential to expand the proteome exponentially, generating a spectrum of activities [16]. Some genes generate tens of thousands of functionally distinct proteins [17]. Splice variants from the same gene can produce proteins with distinct properties and different (even antagonistic) functions [18] [19]. A number of genetic mutations involved in human disease have been mapped to changes in splicing signals or sequences that regulate splicing [20]. In a very relevant context, splice isoform ratios have been proposed to be robust biomarkers for physiological conditions [21]. Thus, an understanding of changes in splicing patterns is critical to a comprehensive understanding of the biological regulation and underlying mechanism of PD.

To date, all of the global expression studies in PD have looked at a single transcript per gene, although most genes have multiple transcripts generated by alternative splicing. Alternative pre-mRNA splicing may be used as a mechanism of gene regulation[22]. The primary long transcript can be processed in many different ways by alternative usage of exons. Regulatory splicing factors, such as SRRM2, influence the expression of numerous pre-mRNAs, often occurring to coordinate changes in protein isoform expression and function. While many modifications resulting from alternative splicing are subtle, they can affect major signaling properties of the nervous system, such as ion channel features, half-life of proteins and gain or loss in receptor function[23]. Multiple intrinsic and environmental stimuli can affect splicing. In addition, splicing errors are associated with a wide variety of diseases including ALS[24], [25], Alzheimer's disease[26], [27], [28] and PD[28], [29], [30], [31], [32], [33]. There is currently little information on the prevalence, type, and significance of splicing of genes in PD in the literature.

The RNA splicing factor SRRM2 which we found as differentially expressed in multiple public PD datasets had alternative splice forms in two brain regions, substantia nigra and amygdala, from PD patients (Figure 2). Our global splicing analysis of blood samples from 17 PD patients and 11 healthy controls showed that SRRM2 has differential exonic expression, with the 5′ exons over-expressed and the 3′ exons down-regulated in PD relative to controls (Figure 3). Interestingly, there were hundreds of genes with significant changes in exonic expression in PD blood versus controls (S2). Currently, we know only a few of the transacting splice factors that control alternative splicing of synaptic protein pre-mRNAs (reviewed in [34]). Future studies aimed at identifying these factors should reveal new signaling pathways that orchestrate changes in alternative splicing of multiple pre-mRNAs to control neuronal excitability and synaptic efficacy.

The final outcome is that differential expression and alternative splicing of SRRM2 potentially invokes high levels of alternative splicing in the genome of PD patients. While experimental validation is needed to support our conclusion, the phenomena of an isoform-dependent regulation of pre-mRNA splicing already exists[35]. While our splicing analysis was done in blood samples, we anticipate that such high levels of alternative splicing, at the transcriptome level, is also present in the brain of PD patients because that the same pattern of SRRM2 splicing and dys-regulation is evident in the substantia nigra and amygdala of PD patients (Figure 2B).

Importantly, the binding of serine arginine proteins such as SRRM2 to their degenerate recognition exonic sequences is intrinsically weak, resulting in a concentration-dependent regulation, for example, certain sequence elements are recognized only at higher SR protein concentration[36]. Therefore, an over-expression of SRRM2 in PD brain and blood may lead to an increased concentration if SRRM2 protein levels enabling slicing events to take place. Further studies will be needed to understand how SRRM2 itself is alternatively spliced and dynamically expressed in PD and to determine whether splicing is prevalent in the substantia nigra of PD patients and how it may play a role in the development of PD.

## Methods

### Analysis of 3 Previous PD Microarray Datasets

We performed extensive analysis on three different publicly available microarray experiments on PD: (A) Twenty-two chips from the substantia nigra in postmortem brain of PD patients and controls [2] (GEO Accession: GSE7621), (B) 21 chips on one to four week rotenone-treated neuroblastoma cells [12], an *in vitro* model of PD (GEO Accession: GSE4773), (C) 105 chips on peripheral blood of PD versus healthy and neurological disease (non-PD) control subjects[11] (GEO Accession: GSE6613). All 151 raw expression files were normalized using GC-RMA processor. All normalized expression data was analyzed using GeneSpring software. Following normalization one-way analysis of variance was performed for each gene to identify statistically significant gene expression changes. Two criteria were used to determine whether a gene was differentially expressed: fold change of ±1.2 and p value <.05 using a two-tailed distribution. No correction for multiple comparisons was done. While a value of 1.2 is considered a low cut-off for fold change in microarrays, it is an accepted cut-off which with statistical significance, is likely to be validated by real-time PCR[37]. Lists of differentially expressed genes from different experiments were compared within GeneSpring and displayed as Venn diagrams to show overlapping and non-overlapping genes (Figure 1). The complete list of overlapping genes is shown in Table S1.

### Real-Time PCR of SRRM2 Isoforms in Postmortem SN and AMG

Tissue samples from 10 sporadic PD patients and 10controls (5 male, 5 female, mean age 84.25-SD 6.4) were examined. Demographics and clinical details for PD patients are provided in Table S5. All controls had normal brain pathology with no evidence or history of neurodegenerative processes. Total RNA was extracted from 100 mg of brain tissue (provided by the University of Miami Brain Bank) using TRIzol Reagent (Invitrogen, Carlsbad, CA). For each extraction RNA concentration and integrity was determined by an Agilent Nanodrop Spectrophotometer and Agilent BioAnalyzer respectively, and cDNA was synthesized using high capacity cDNA kit (Applied Biosystems, Foster City, CA). SRRM2 expression level was measured in each sample by real-time PCR using the ABI 7900HT thermocycler. SRRM2 expression levels were normalized to those of the internal reference 18S rRNA (Hs99999901_s1). All samples were run in duplicates. cDNA was amplified using TaqMan Universal PCR master mix reagent (Applied Biosystems, Foster City, CA) at the following conditions: 10 minutes at 25°C, 120 minutes at 37°C, and 5 seconds at 85°C. The two target SRRM2 cDNAs were amplified using TaqMan assay (∼120 bp sequence) Hs00909897_m1, interrogating exon 2–3 boundary, and Hs00249492_m1, interrogating exons 12–13 boundary. Data was analyzed using software RQ manager 1.2 from Applied Biosystems, CA.

### Subject Recruitment

Human subjects were recruited at the outpatients department of the Movement Disorders Division of the Department of Neurology, University of Miami, Miller School of Medicine. The Institutional Review Board of The University of Miami approved this study. Written informed consent was obtained from all study participants. Whole blood samples were collected under approved ethical committee protocols from the Movement Disorders Division of the Department of Neurology, University of Miami, Miller School of Medicine. All patients were diagnosed by at least 2 board-certified and fellowship-trained movement disorders neurologists according to the UK Society Brain Bank Criteria for the diagnosis of PD [38]. Control subjects were spouses or caregivers of patients who had no personal or family history of neurodegenerative diseases. Exclusion criteria for all study subjects were age under 21 years, hematologic malignancies or coagulopathies, known severe anemia (hematocrit >30), and known pregnancy. Clinical data of subjects is summarized in Table S6.

### RNA Isolation for Exon Arrays

RNA isolation was performed using the Preanalytix Paxgene blood RNA kit, and small RNAs were extracted using a modified RNEasy protocol. Paxgene blood RNA tubes were centrifuged to form a pellet. Then an enzymatic digestion step followed by a filtration step was used to remove gDNA and cell debris. Ethanol was added to the lysate and passed through a silica membrane. The flow through from this step was collected for use in the small RNA extraction. After a series of washes and a DNase step, RNA was eluted in 60 µL of RNase/DNase free water. Then 1.4x volumes of ethanol was added to the collected flow through and passed through an RNeasy silica gel membrane. Small RNAs were eluted in 40 µL of RNAse/DNase free water. RNA was analyzed and quantified by digital electrophoresis (Agilnet Bioanalyzer, Agilent Technologies) and UV spectrophotometry (Nanodrop 1000, Thermo Scientific). 28 (17 PDs and 11 controls) samples passed the quality control (RIN>5) and concentration requirement for the Exon microarrays, while 21 samples (13 PDs and 8 controls) passed those requirements for the miRNA TLDA cards. All the RNA samples used in our expression study had RIN values between 7 and 9.

### Exon Arrays: Global Transcription Analysis and Splicing Analysis

1 microgram of RNA (excluding small RNAs) from each of the 28 subjects (17 PDs and 11 controls) was labeled for hybridization to Affymetrix Human Exon Arrays covering known cDNAs, ESTs, and predicted gene structure sequences, using standard Affymetrix protocols. Briefly, labeled samples were added to arrays, and arrays were hybridized for 17 hours at 45°C. The arrays were stained and washed according to Affymetrix Fluidics Station 450 protocol (FS450_0001). Hybridization was documented using a GeneChip Scanner 3000 7G and validated with Affymetrix Microarray Suite version 5.0 (MAS 5.0) software. Pearson correlation coefficients demonstrated high reproducibility. Two samples (CT#3, PD#19) were acting as outliers and were therefore excluded from the analysis. Subsequent statistical analysis was performed using GeneSpring GX 10 software (Silicon Genetics, Redwood City, CA). Normalized expression values were calculated by the GC-Robust Multi-array Average (GC-RMA) method. The resultant signal information was analyzed using one-way analysis of variance (ANOVA) (*p<0.05*), assuming normality and equal variances. No correction for multiple comparisons was done. GeneSpring's cross gene error model, which determines the likelihood of observing a specific fold change to the likelihood of observing a fold change of 1, was active during this test. Hierarchical clustering analysis was performed using the GeneSpring 10 software (Silicon Genetics, Inc., Redwood city, CA, USA) to generate dendrograms representing each functional category of genes based on their expression profiles. Heat maps were generated by dividing each measurement by the 50.0th percentile of all measurements in that sample, then setting the average value of expression level for each gene across the samples to 1.0, and plotting the resulting normalized signal value for each sample (values below 0.01 were set to 0.01). The list and the order of various genes in which they appear in the heatmaps can be viewed in tabular form.

For splicing analysis, core transcripts were normalized via RMA16 and filtered for detection above background level (*p<0.00025*). Splicing ANOVA was then performed with a *p<0.05* and a Benjamini Hochberg FDR correction. The resulting transcripts were further filtered by a splicing index of 0.87 to yield a total of 218 transcripts with exons with statistically significant changes in inclusion rates (relative to the gene level) between PDs and controls. The output of 218 alternatively spliced transcripts were subjected to Gene Ontology (GO) analysis within GeneSpring X to find any significant over-representation of molecular category. A statistical test assigns a p-value to each category. All data is MIAME compliant and all raw.CEL files from the 28 exon arrays are deposited in NCBI's GEO database (GSE 18838), a MIAMI compliant database.

For confirmation of gender in our cohort, we looked at the expression levels of 18 Y-linked genes and confirmed that they were highly expressed in the male subjects. (See Supplementary Figure S2).

## Supporting Information

Figure S1 Stratifying microarray data by gender. Venn diagrams show genes differentially expressed (p<.05) by 1.2 fold (A) or 2.0 fold (B) in females only, males only, or both females and males. No multiple correction was done.
(6.06 MB TIF)

Figure S2 Confirmation of gender of microarray cohort by expression of 18 Y-linked genes. Box plot shows average expression levels of 18 Y-linked genes in each chip. Males show high expression of Y chromosome genes. The 18 genes used are RPS4Y1,ZFY, PCDH11Y, TBL1Y, PRKY, TTTY12, USP9Y, TMSB4Y, NLGN4Y, RPS4Y2, RBMY1F, PRY, AMELY, TTTY11, TTTY14, TTTY10, TTTY13, TTTY5.
(5.50 MB TIF)

Table S1 Differentially expressed genes overlapping among 3 PD microarray datasets. Listed are genes differentially expressed by 1.2 fold (p<.05 with no multiple correction) in any 2 of the 3 PD public datasets analyzed.
(0.04 MB XLS)

Table S2 Transcripts with differential exonic expression in PD blood. Affymetrix Exon_ST1 arrays showed 218 transcripts with significant change (p<0.05 with Benjamini Hochberg FDR correction) in exonic expression in 17 PD blood samples versus 11 healthy controls.
(0.24 MB DOC)

Table S3 Transcripts with significant exonic expression in PD blood show over- representation of Protein Binding Gene Ontology (GO) function. 112/218 transcripts with significant change in exonic expression in 17 PD blood samples versus healthy 11 controls belong to the GO Biological Function Protein Binding.
(0.12 MB DOC)

Table S4 Genes differentially regulated by at least 2.0 fold in PD blood. 58 genes were differerntially expressed (FC > = 2.0; p<.05 with no multiple correction) in the blood of 17 PDs versus 11 controls.
(0.03 MB XLS)

Table S5 Characteristics of our PD cohort included in our Real-Time PCR analysis. M =  Male, F =  Female, A–R =  Akinetic-Rigid dominant PD, Tremor =  Tremor dominant PD, HY =  Hoehn and Yahr Parkinson's disease stage, PMI =  post-mortem interval.
(0.05 MB DOC)

Table S6 Characteristics of our PD and control cohort included in our Affymetrix Exon Arrays and TaqMan Low Density Arrays. Control and PD subjects were recruited for our study. The best quality blood RNA used on the arrays came from 17 PDs and 11 controls.
(0.03 MB XLS)
