# Supplementary figures and images for: SRRM2, a Potential Blood Biomarker Revealing High Alternative Splicing in Parkinson's Disease
Source: PLoS One. 2010 Feb 8;5(2):e9104. doi: 10.1371/journal.pone.0009104 (PMC2817002; doi:10.1371/journal.pone.0009104)

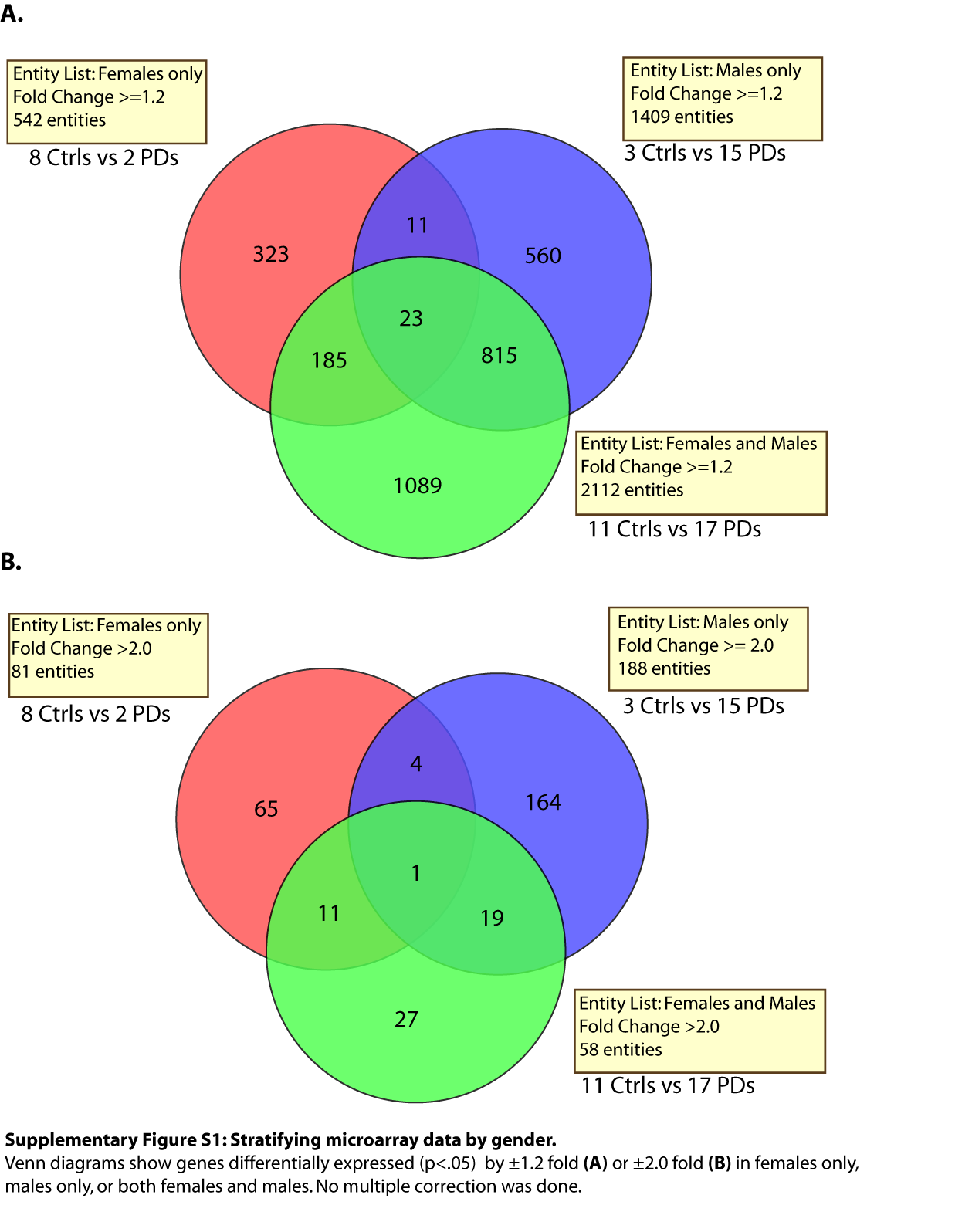

Supplement: Figure S1 — Stratifying microarray data by gender. Venn diagrams show genes differentially expressed (p<.05) by 1.2 fold (A) or 2.0 fold (B) in females only, males only, or both females and males. No multiple correction was done. (6.06 MB TIF) [file pone.0009104.s001.tif]

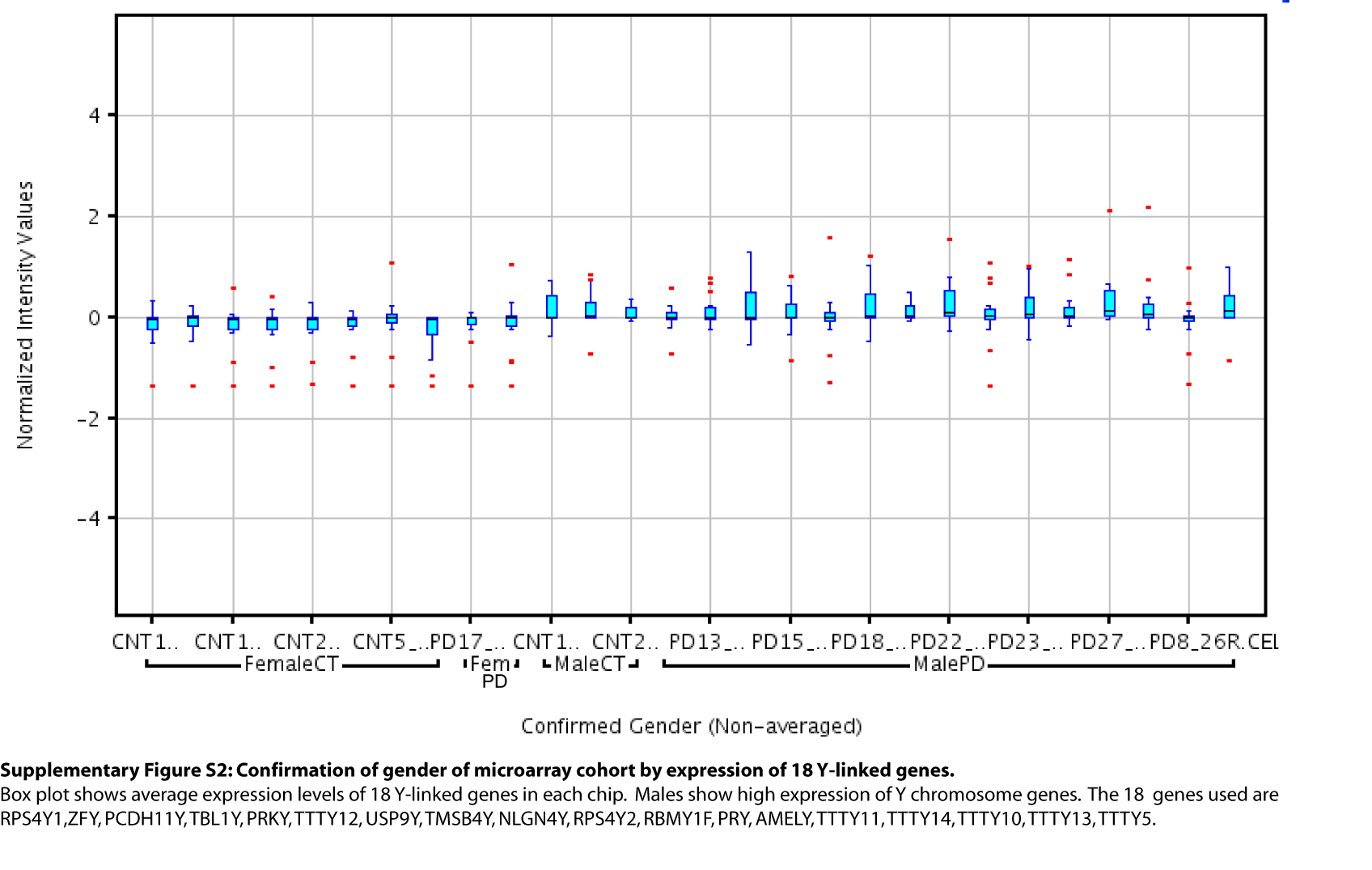

Supplement: Figure S2 — Confirmation of gender of microarray cohort by expression of 18 Y-linked genes. Box plot shows average expression levels of 18 Y-linked genes in each chip. Males show high expression of Y chromosome genes. The 18 genes used are RPS4Y1,ZFY, PCDH11Y, TBL1Y, PRKY, TTTY12, USP9Y, TMSB4Y, NLGN4Y, RPS4Y2, RBMY1F, PRY, AMELY, TTTY11, TTTY14, TTTY10, TTTY13, TTTY5. (5.50 MB TIF) [file pone.0009104.s002.tif]
