# Supplementary material for: SRRM2, a Potential Blood Biomarker Revealing High Alternative Splicing in Parkinson's Disease
Source: PLoS One. 2010 Feb 8;5(2):e9104. doi: 10.1371/journal.pone.0009104 (PMC2817002; doi:10.1371/journal.pone.0009104)
Supplement: Table S2 — Transcripts with differential exonic expression in PD blood. Affymetrix Exon_ST1 arrays showed 218 transcripts with significant change (p<0.05 with Benjamini Hochberg FDR correction) in exonic expression in 17 PD blood samples versus 11 healthy controls. (0.24 MB DOC) [file pone.0009104.s004.doc]

| **Transcripts Cluster Id** | **Splicing Index [PD] vs [CNT]** | **genbank** | **refseq** | **genesymbol** |
| --- | --- | --- | --- | --- |
| 2320727 | 1.0754642 | BC052977 | NM_001066 | TNFRSF1B |
| 2321779 | 1.079116 | BC007233 | NM_024329 | EFHD2 |
| 2322389 | 1.211742 | AK024468 | NM_018090 | NECAP2 |
| 2326463 | 1.2004877 | BC000644 | NM_001803 | CD52 |
| 2327283 | -0.87658215 | AF044896 | NM_001105556 | C1orf38 |
| 2351854 | 0.9134476 | BC017973 |  | C1orf162 |
| 2352228 | 0.8944664 | BX648738 | NM_006135 | CAPZA1 |
| 2358906 | 1.1360856 | S71381 | NM_002796 | PSMB4 |
| 2383726 | 1.2687463 | BC011358 | NM_001024226 | ARF1 |
| 2395490 | 0.91319585 | BC011130 | NM_001428 | ENO1 |
| 2402111 | -0.8967414 | AK292532 | NM_020317 | C1orf63 |
| 2403111 | 1.1016101 | BC040943 | NM_006990 | WASF2 |
| 2412360 | -0.89790463 | U07707 | NM_001981 | EPS15 |
| 2412668 | -1.2123625 | AK056169 | NM_015913 | TXNDC12 |
| 2416522 | 0.9736277 | BC132729 | NM_002227 | JAK1 |
| 2419235 | 1.155994 | AB209366 | NM_003902 | FUBP1 |
| 2420467 | -0.9242613 | BC024007 | NM_004388 | CTBS |
| 2422035 | 1.010676 | AK090479 | NM_052942 | GBP5 |
| 2427619 | -0.90107775 | M85217 | NM_002232 | KCNA3 |
| 2434438 | -0.92358327 | BC017197 | NM_021960 | MCL1|C1orf138 |
| 2434575 | -0.91337323 | BC002642 | NM_004079 | CTSS |
| 2440354 | -0.9399495 | BC016182 | NM_001778 | CD48 |
| 2447414 | 1.1830003 | AB209647 | NM_000433 | NCF2 |
| 2447454 | 0.9876499 | AK095424 | NM_005717 | ARPC5 |
| 2448073 | 0.91640997 | AF205218 | NM_006469 | IVNS1ABP |
| 2451463 | 1.1293354 | BC001594 | NM_015999 | ADIPOR1 |
| 2464499 | 1.1226878 | BC003367 | NM_031844 | HNRNPU |
| 2473149 | 1.0807396 | BC111534 | NM_147223 | NCOA1 |
| 2488732 | 1.0541373 | BC088351 | NM_006429 | CCT7 |
| 2496628 | 1.1141886 | BC064421 |  | C2orf29 |
| 2496727 | 0.91751087 | AY212247 | NM_145686 | MAP4K4 |
| 2499053 | -1.3376276 | AF527760 | NM_004987 | LIMS1 |
| 2534354 | 0.9646241 | AF115510 | NM_004735 | LRRFIP1 |
| 2540157 | 0.87168854 | AK292352 | NM_002539 | ODC1 |
| 2542816 | 0.9102407 | AF315591 | NM_015317 | PUM2 |
| 2577958 | -1.1119467 | J05032 | NM_001349 | DARS |
| 2578028 | 0.9170045 | AF147204 | NM_001008540 | CXCR4 |
| 2591837 | 0.88806915 | AF215636 | NM_014585 | SLC40A1 |
| 2603051 | -1.3788798 | AF280095 | NM_080424 | SP110 |
| 2608765 | 0.9687494 | BC063125 | NM_018184 | ARL8B |
| 2609608 | 0.9762502 | AB051544 | NM_001080517 | SETD5 |
| 2624110 | -0.9739093 | BC000884 | NM_014041 | SPCS1 |
| 2628682 | -0.9256943 | AF070523 | NM_006407 | ARL6IP5 |
| 2639054 | 1.0621316 | BX648758 | NM_017554 | PARP14 |
| 2640916 | 0.88653827 | BC011858 | NM_172027 | ABTB1 |
| 2647458 | 0.9751172 | BC009803 | NM_007282 | RNF13 |
| 2648677 | 1.0362827 | J03779 | NM_007288 | MME |
| 2663244 | 0.9761485 | BC018119 | NM_002880 | RAF1 |
| 2674179 | 0.89641255 | BC125130 | NM_003363 | USP4 |
| 2674242 | -1.546423 | BX647063 | NM_001664 | RHOA |
| 2676671 | -1.0629336 | BX647248 | NM_001064 | TKT |
| 2681753 | 0.95183325 | AK122710 | NM_032682 | FOXP1 |
| 2685034 | -0.9512472 | BC052980 | NM_001008390 | CGGBP1 |
| 2694397 | 0.88169324 | BC010839 | NM_002950 | RPN1 |
| 2702307 | -1.6315129 | BC038394 | NM_020307 | CCNL1 |
| 2712040 | 1.1765504 | BC060767 | NM_012287 | CENTB2 |
| 2714818 | 0.87611187 | BC131709 | NM_175918 | CRIPAK |
| 2715634 | 0.92943865 | BC042998 | NM_176801 | ADD1 |
| 2735459 | 0.885536 | AK289990 | NM_014606 | HERC3 |
| 2748198 | 0.97379214 | BC131505 | NM_015196 | KIAA0922 |
| 2761285 | -0.9733212 | BC016987 | NM_148894 | FAM44A |
| 2774365 | 1.0147115 | BC004975 | NM_006835 | CCNI |
| 2775756 | 1.0727487 | AB018359 | NM_014933 | SEC31A |
| 2784027 | -0.8954439 | BC018671 | NM_001154 | ANXA5 |
| 2816298 | 0.9347682 | AK291066 | NM_006633 | IQGAP2 |
| 2868131 | -0.9144063 | AF222340 | NM_001040458 | ERAP1 |
| 2878437 | -1.1342504 | BC010507 | NM_001040021 | CD14 |
| 2891052 | 0.9623779 | BC010119 | NM_006098 | GNB2L1 |
| 2899372 | -0.96519756 | AK290193 | NM_007048 | BTN3A1 |
| 2902427 | 0.9572376 | AF000424 | NM_007161 | LST1 |
| 2903401 | 1.4542248 | AY831402 | NM_002121 | HLA-DPB1 |
| 2905256 | 0.8738489 | BC095423 | NM_152734 | C6orf89 |
| 2907190 | 1.0778887 | AY061884 | NM_015255 | UBR2 |
| 2907459 | 0.96144265 | AK090425 | NM_006586 | CNPY3 |
| 2929168 | 1.126455 | AK292506 | NM_007124 | UTRN |
| 2934308 | 1.0601821 | J03528 | NM_000876 | IGF2R |
| 2945741 | 1.0678964 | U49187 | NM_014722 | C6orf32 |
| 2950329 | -1.0604779 | AK292709 | NM_033554 | HLA-DPA1 |
| 2957596 | 0.88175476 | AK125098 | NM_021814 | ELOVL5 |
| 2960903 | 0.9333595 | BC019669 | NM_001402 | EEF1A1 |
| 2966253 | 1.0712837 | AF314184 | NM_032870 | SFRS18 |
| 2969886 | 1.1810914 | M14333 | NM_002037 | FYN |
| 2974635 | 0.9159889 | D89974 | NM_004665 | VNN2 |
| 2982076 | 0.8727275 | AF385429 | NM_054114 | TAGAP |
| 3014714 | -1.400178 | AF006084 | NM_005720 | ARPC1B |
| 3028977 | -1.020609 | AK095263 | NM_015917 | GSTK1 |
| 3032017 | 0.8763375 | AF459743 | NM_016118 | NUB1 |
| 3037193 | 0.9139078 | AB037790 | NM_014413 | EIF2AK1 |
| 3057955 | 1.0856516 | BC033820 | NM_006682 | FGL2 |
| 3066297 | 1.0275123 | U88666 | NM_182691 | SRPK2 |
| 3076178 | -1.0581737 | AK127030 | NM_013446 | MKRN1 |
| 3099750 | -0.88816243 | AK128645 | NM_005625 | SDCBP |
| 3126087 | 0.9775075 | BC016481 | NM_177924 | ASAH1 |
| 3131741 | 0.94219214 | AY280968 | NM_001002814 | RAB11FIP1 |
| 3154263 | 1.0798551 | D89077 | NM_001045556 | SLA |
| 3178952 | -0.87536144 | BC002962 | NM_003177 | SYK |
| 3186966 | 1.0478239 | AK290053 | NM_138554 | TLR4 |
| 3193339 | 0.9230457 | BC063827 | NM_002957 | RXRA |
| 3204404 | -0.88700074 | BC122550 | NM_007126 | VCP |
| 3217736 | -1.0166277 | BX648519 | NM_015051 | TXNDC4 |
| 3229338 | -1.1075902 | BC020635 | NM_002003 | FCN1 |
| 3235461 | 0.88327736 | AK225634 | NM_006023 | CDC123 |
| 3236958 | -1.2927563 | BC066956 | NM_003380 | VIM |
| 3239760 | 0.9521965 | BC054516 | NM_019043 | APBB1IP |
| 3255220 | 0.90745056 | AF131820 | NM_014394 | GHITM |
| 3256689 | -0.9581946 | U92436 | NM_000314 | PTEN |
| 3268669 | 1.0716488 | AF047472 | NM_004725 | BUB3 |
| 3284188 | 1.0569562 | AK291697 | NM_002211 | ITGB1 |
| 3291682 | -1.0321306 | EF068222 | NM_032776 | JMJD1C |
| 3293762 | -1.0785252 | BC004275 | NM_002778 | PSAP |
| 3297536 | 1.0259556 | AK126287 | NM_145869 | ANXA11 |
| 3301914 | 0.8799088 | AK122584 | NM_152309 | PIK3AP1 |
| 3302187 | 1.1848602 | AK090447 | NM_032900 | ARHGAP19 |
| 3304624 | 1.0190073 | BX648078 | NM_012229 | NT5C2 |
| 3325680 | 0.9066311 | AK292139 | NM_006360 | EIF3M |
| 3326400 | 0.9952993 | BC110398 | NM_001752 | CAT |
| 3340697 | 0.89583254 | AB012958 | NM_003369 | UVRAG |
| 3393670 | -1.0882763 | AK090409 | NM_001098526 | AMICA1 |
| 3393744 | -1.0044608 | BC039035 | NM_000732 | CD3D |
| 3396916 | 0.97486687 | BC001162 | NM_003139 | SRPR |
| 3403092 | -0.90523076 | M77273 | NM_080549 | PTPN6 |
| 3404030 | 1.0219766 | BC012621 | NM_005810 | KLRG1 |
| 3404436 | 0.90002763 | BC019883 | NM_001004419 | CLEC2D |
| 3408505 | -1.0881295 | BC126417 | NM_006152 | LRMP |
| 3414186 | 1.1954086 | AK124849 | NM_001098576 | TEGT |
| 3414739 | -0.9748168 | BC004492 | NM_014033 | METTL7A |
| 3414846 | 1.0587027 | AK125855 | NM_014764 | DAZAP2 |
| 3422855 | 1.3538411 | BC012510 | NM_006851 | GLIPR1 |
| 3431376 | 0.91761565 | BC032833 | NM_033121 | ANKRD13A |
| 3434413 | 0.91044104 | AB027196 | NM_014868 | RNF10 |
| 3439603 | 1.1548008 | AF007135 | NM_005056 | JARID1A |
| 3445544 | -0.875993 | AK290914 | NM_024829 | FLJ22662 |
| 3453837 | -1.1973127 | AF141347 | NM_006009 | TUBA1A |
| 3457160 | -0.95163715 | M59907 | NM_001780 | CD63 |
| 3458337 | 1.0758402 | BC075852 | NM_003153 | STAT6 |
| 3460593 | -1.1470083 | AL117550 | NM_016056 | TMBIM4 |
| 3463571 | 0.8938299 | AF458589 | NM_002480 | PPP1R12A |
| 3464860 | -0.9744129 | BC005047 | NM_001946 | DUSP6 |
| 3465409 | 0.96206975 | BC009050 | NM_001731 | BTG1|LOC256021 |
| 3473083 | 1.0997076 | AY338463 | NM_015335 | MED13L |
| 3473802 | 1.1237332 | AF181985 | NM_016281 | TAOK3 |
| 3477917 | 0.9238972 | AY038999 | NM_145648 | SLC15A4 |
| 3498502 | 0.8857094 | BC110656 | NM_004800 | TM9SF2 |
| 3528805 | 1.0443103 | BC112331 | NM_005015 | OXA1L |
| 3529609 | -1.0858842 | L07633 | NM_176783 | PSME1 |
| 3536663 | 0.9175942 | BC015621 | NM_144578 | MAPK1IP1L |
| 3556556 | 0.9167007 | BC009798 | NM_001344 | DAD1|OR6J1 |
| 3564210 | 1.0108463 | BC095850 | NM_002863 | PYGL |
| 3569754 | 0.9343362 | X79067 | NM_004926 | ZFP36L1 |
| 3569814 | 0.9883629 | DQ496098 | NM_001102 | ACTN1 |
| 3571347 | 1.1651292 | AF171938 | NM_001005743 | NUMB |
| 3571904 | -0.92335534 | BC002532 | NM_006432 | NPC2 |
| 3587015 | 0.9010033 | AF132599 | NM_015995 | KLF13 |
| 3592023 | -1.2394958 | AK026463 | NM_004048 | B2M |
| 3606304 | 1.0078758 | AF406992 | NM_006738 | AKAP13 |
| 3609138 | 0.9641635 | AF006514 | NM_001271 | CHD2 |
| 3617312 | 1.2635942 | BC051744 | NM_001042496 | SLC12A6 |
| 3644973 | -1.0539606 | BC033494 | NM_002613 | PDPK1 |
| 3645253 | 0.8766563 | AB016092 | NM_016333 | SRRM2 |
| 3651955 | -0.89581376 | BC000195 | NM_016025 | METTL9 |
| 3653123 | 0.99378395 | M13975 | NM_002738 | PRKCB1 |
| 3657041 | 0.92566156 | M81695 | NM_000887 | ITGAX |
| 3661065 | -0.9195955 | BC034490 | NM_005611 | RBL2 |
| 3679564 | 0.9353883 | AY376241 | NM_003470 | USP7 |
| 3680434 | 0.889231 | AK095955 | NM_004862 | LITAF |
| 3680953 | -1.1960146 | AY553877 | NM_018340 | FLJ11151 |
| 3683050 | 1.1028936 | AB061371 | NM_015092 | SMG1 |
| 3695699 | 0.94400895 | BC008861 | NM_004691 | ATP6V0D1 |
| 3708422 | -1.0313685 | AY129319 | NM_001970 | EIF5A |
| 3716411 | 0.9447923 | U65090 | NM_001304 | CPD |
| 3722917 | 1.0853271 | AF055008 | NM_002087 | GRN |
| 3724698 | 0.8835809 | BC065294 | NM_006310 | NPEPPS |
| 3737274 | 1.0365086 | BC036891 |  | KIAA1618 |
| 3742783 | 1.0125878 | AF310105 | NM_033004 | NLRP1 |
| 3752258 | 0.8766375 | BC005926 | NM_006495 | EVI2B |
| 3753860 | 0.92179424 | M21121 | NM_002985 | CCL5 |
| 3756319 | 0.94952565 | L31581 | NM_001838 | CCR7 |
| 3759006 | -0.9135158 | BC096107 | NM_000342 | SLC4A1 |
| 3759077 | 0.90423924 | AB209576 | NM_016016 | SLC25A39 |
| 3770743 | 0.97544277 | AK091010 | NM_002086 | GRB2 |
| 3773932 | -1.0660604 | BC009848 | NM_001614 | ACTG1 |
| 3786039 | 1.079606 | BC033004 | NM_002647 | PIK3C3 |
| 3806913 | 0.88418293 | BC014840 | NM_005901 | SMAD2 |
| 3816380 | -2.0100503 | D87914 | NM_004152 | OAZ1 |
| 3819543 | 0.8755927 | BC019580 | NM_005968 | HNRNPM |
| 3824874 | -1.239711 | AK123477 | NM_006332 | IFI30|PIK3R2 |
| 3834089 | 1.0149999 | BC002564 | NM_007040 | HNRNPUL1 |
| 3837731 | 0.91148794 | BC009718 | NM_001425 | EMP3 |
| 3838385 | 0.8727226 | AL833693 | NM_001774 | CD37 |
| 3841076 | -1.2244453 | BC095412 | NM_001020818 | MYADM |
| 3842141 | -0.9354322 | AB209627 | NM_000991 | RPL28 |
| 3844855 | 1.0758083 | BC012775 |  | C19orf22 |
| 3846538 | -0.99401844 | BC126259 | NM_001961 | EEF2 |
| 3846783 | 1.3116627 | AF272894 | NM_025241 | UBXD1 |
| 3850261 | -1.3393446 | BC046121 | NM_002162 | ICAM3 |
| 3852832 | 1.1160908 | AF239764 | NM_032571 | EMR3 |
| 3860137 | -0.89598095 | BC011175 | NM_003332 | TYROBP |
| 3861581 | 0.9919495 | BC011792 | NM_001398 | ECH1 |
| 3869237 | -0.91055983 | BC005315 | NM_002029 | FPR1 |
| 3883382 | 1.0742778 | EF206690 | NM_198398 | ERGIC3 |
| 3886704 | 1.1210654 | BC093768 | NM_006282 | STK4 |
| 3887117 | 1.0178142 | AK172808 | NM_000308 | CTSA |
| 3887635 | 0.99289346 | AF012108 | NM_181659 | NCOA3 |
| 3890109 | 0.8769494 | BC017725 | NM_080821 | C20orf108 |
| 3908358 | 0.90547323 | AB033073 | NM_018837 | SULF2 |
| 3908831 | 0.89643216 | AK002139 | NM_021035 | ZNFX1 |
| 3920850 | 0.9425228 | BC013327 | NM_002243 | KCNJ15 |
| 3942648 | 0.93390393 | BC037986 | NR_002323 | TUG1 |
| 3942838 | 1.0218909 | BC013051 | NM_016733 | LIMK2 |
| 3960629 | 0.97738683 | BC000595 | NM_006386 | DDX17 |
| 3971806 | -0.99396545 | AL050290 | NM_002970 | SAT1 |
| 3971877 | 0.8868539 | BC019906 | NM_001415 | EIF2S3 |
| 3973839 | 0.9233717 | BC032720 | NM_000397 | CYBB |
| 3980887 | -1.0969043 | BC028299 | NM_007363 | NONO |
| 3987029 | 0.90128094 | AK026332 | NM_032227 | TMEM164 |
| 4022106 | 1.048109 | AY072692 | NM_018388 | MBNL3 |
| 4025339 | 1.1237578 | DQ160194 | NM_000202 | IDS |
| 4037656 | -1.0008075 | AF253979 | NC_001807 | ND4L |

**Supplementary Table S2: Transcripts with differential exonic expression in PD blood.** Affymetrix Exon_ST1 arrays showed 218 transcripts with significant change (*p<0.05* with Benjamini Hochberg FDR correction) in exonic expression in 17 PD blood samples versus 11 healthy controls.
