# Supplementary material for: SRRM2, a Potential Blood Biomarker Revealing High Alternative Splicing in Parkinson's Disease
Source: PLoS One. 2010 Feb 8;5(2):e9104. doi: 10.1371/journal.pone.0009104 (PMC2817002; doi:10.1371/journal.pone.0009104)
Supplement: Table S3 — Transcripts with significant exonic expression in PD blood show over- representation of Protein Binding Gene Ontology (GO) function. 112/218 transcripts with significant change in exonic expression in 17 PD blood samples versus healthy 11 controls belong to the GO Biological Function Protein Binding. (0.12 MB DOC) [file pone.0009104.s005.doc]

| **transcript_cluster_id** | **genbank** | **refseq** | **genesymbol** |
| --- | --- | --- | --- |
| 2320727 | BC052977 | NM_001066 | TNFRSF1B |
| 2322389 | AK024468 | NM_018090 | NECAP2 |
| 2352228 | BX648738 | NM_006135 | CAPZA1 |
| 2383726 | BC011358 | NM_001024226 | ARF1 |
| 2395490 | BC011130 | NM_001428 | ENO1 |
| 2403111 | BC040943 | NM_006990 | WASF2 |
| 2412360 | U07707 | NM_001981 | EPS15 |
| 2416522 | BC132729 | NM_002227 | JAK1 |
| 2419235 | AB209366 | NM_003902 | FUBP1 |
| 2427619 | M85217 | NM_002232 | KCNA3 |
| 2434438 | BC017197 | NM_021960 | MCL1 |
| 2440354 | BC016182 | NM_001778 | CD48 |
| 2447414 | AB209647 | NM_000433 | NCF2 |
| 2447454 | AK095424 | NM_005717 | ARPC5 |
| 2448073 | AF205218 | NM_006469 | IVNS1ABP |
| 2464499 | BC003367 | NM_031844 | HNRNPU |
| 2488732 | BC088351 | NM_006429 | CCT7 |
| 2534354 | AF115510 | NM_004735 | LRRFIP1 |
| 2540157 | AK292352 | NM_002539 | ODC1 |
| 2542816 | AF315591 | NM_015317 | PUM2 |
| 2577958 | J05032 | NM_001349 | DARS |
| 2578028 | AF147204 | NM_001008540 | CXCR4 |
| 2603051 | AF280095 | NM_080424 | SP110 |
| 2608765 | BC063125 | NM_018184 | ARL8B |
| 2628682 | AF070523 | NM_006407 | ARL6IP5 |
| 2640916 | BC011858 | NM_172027 | ABTB1 |
| 2647458 | BC009803 | NM_007282 | RNF13 |
| 2648677 | J03779 | NM_007288 | MME |
| 2663244 | BC018119 | NM_002880 | RAF1 |
| 2674242 | BX647063 | NM_001664 | RHOA |
| 2676671 | BX647248 | NM_001064 | TKT |
| 2694397 | BC010839 | NM_002950 | RPN1 |
| 2714818 | BC131709 | NM_175918 | CRIPAK |
| 2715634 | BC042998 | NM_176801 | ADD1 |
| 2775756 | AB018359 | NM_014933 | SEC31A |
| 2784027 | BC018671 | NM_001154 | ANXA5 |
| 2868131 | AF222340 | NM_001040458 | ERAP1 |
| 2878437 | BC010507 | NM_001040021 | CD14 |
| 2891052 | BC010119 | NM_006098 | GNB2L1 |
| 2902427 | AF000424 | NM_007161 | LST1 |
| 2907190 | AY061884 | NM_015255 | UBR2 |
| 2929168 | AK292506 | NM_007124 | UTRN |
| 2934308 | J03528 | NM_000876 | IGF2R |
| 2960903 | BC019669 | NM_001402 | EEF1A1 |
| 2969886 | M14333 | NM_002037 | FYN |
| 3014714 | AF006084 | NM_005720 | ARPC1B |
| 3037193 | AB037790 | NM_014413 | EIF2AK1 |
| 3066297 | U88666 | NM_182691 | SRPK2 |
| 3076178 | AK127030 | NM_013446 | MKRN1 |
| 3099750 | AK128645 | NM_005625 | SDCBP |
| 3131741 | AY280968 | NM_001002814 | RAB11FIP1 |
| 3154263 | D89077 | NM_001045556 | SLA |
| 3178952 | BC002962 | NM_003177 | SYK |
| 3186966 | AK290053 | NM_138554 | TLR4 |
| 3193339 | BC063827 | NM_002957 | RXRA |
| 3204404 | BC122550 | NM_007126 | VCP |
| 3217736 | BX648519 | NM_015051 | TXNDC4 |
| 3229338 | BC020635 | NM_002003 | FCN1 |
| 3236958 | BC066956 | NM_003380 | VIM |
| 3256689 | U92436 | NM_000314 | PTEN |
| 3268669 | AF047472 | NM_004725 | BUB3 |
| 3284188 | AK291697 | NM_002211 | ITGB1 |
| 3291682 | EF068222 | NM_032776 | JMJD1C |
| 3297536 | AK126287 | NM_145869 | ANXA11 |
| 3302187 | AK090447 | NM_032900 | ARHGAP19 |
| 3325680 | AK292139 | NM_006360 | EIF3M |
| 3393670 | AK090409 | NM_001098526 | AMICA1 |
| 3393744 | BC039035 | NM_000732 | CD3D |
| 3403092 | M77273 | NM_080549 | PTPN6 |
| 3404436 | BC019883 | NM_001004419 | CLEC2D |
| 3414846 | AK125855 | NM_014764 | DAZAP2 |
| 3434413 | AB027196 | NM_014868 | RNF10 |
| 3439603 | AF007135 | NM_005056 | JARID1A |
| 3458337 | BC075852 | NM_003153 | STAT6 |
| 3460593 | AL117550 | NM_016056 | TMBIM4 |
| 3463571 | AF458589 | NM_002480 | PPP1R12A |
| 3465409 | BC009050 | NM_001731 | BTG1|LOC256021 |
| 3569754 | X79067 | NM_004926 | ZFP36L1 |
| 3569814 | DQ496098 | NM_001102 | ACTN1 |
| 3571347 | AF171938 | NM_001005743 | NUMB |
| 3571904 | BC002532 | NM_006432 | NPC2 |
| 3587015 | AF132599 | NM_015995 | KLF13 |
| 3592023 | AK026463 | NM_004048 | B2M |
| 3606304 | AF406992 | NM_006738 | AKAP13 |
| 3644973 | BC033494 | NM_002613 | PDPK1 |
| 3645253 | AB016092 | NM_016333 | SRRM2 |
| 3657041 | M81695 | NM_000887 | ITGAX |
| 3661065 | BC034490 | NM_005611 | RBL2 |
| 3680434 | AK095955 | NM_004862 | LITAF |
| 3683050 | AB061371 | NM_015092 | SMG1 |
| 3708422 | AY129319 | NM_001970 | EIF5A |
| 3724698 | BC065294 | NM_006310 | NPEPPS |
| 3742783 | AF310105 | NM_033004 | NLRP1 |
| 3759006 | BC096107 | NM_000342 | SLC4A1 |
| 3773932 | BC009848 | NM_001614 | ACTG1 |
| 3786039 | BC033004 | NM_002647 | PIK3C3 |
| 3806913 | BC014840 | NM_005901 | SMAD2 |
| 3816380 | D87914 | NM_004152 | OAZ1 |
| 3819543 | BC019580 | NM_005968 | HNRNPM |
| 3824874 | AK123477 | NM_006332 | IFI30 |
| 3834089 | BC002564 | NM_007040 | HNRNPUL1 |
| 3842141 | AB209627 | NM_000991 | RPL28 |
| 3846538 | BC126259 | NM_001961 | EEF2 |
| 3860137 | BC011175 | NM_003332 | TYROBP |
| 3861581 | BC011792 | NM_001398 | ECH1 |
| 3883382 | EF206690 | NM_198398 | ERGIC3 |
| 3886704 | BC093768 | NM_006282 | STK4 |
| 3887117 | AK172808 | NM_000308 | CTSA |
| 3887635 | AF012108 | NM_181659 | NCOA3 |
| 3942838 | BC013051 | NM_016733 | LIMK2 |
| 3971877 | BC019906 | NM_001415 | EIF2S3 |
| 4037656 | AF253979 | NC_001807 | ND4L |

**Supplementary Table S3: Transcripts with significant exonic expression in PD blood show over- representation of Protein Binding Gene Ontology (GO) function.** 112/218 transcripts with significant change in exonic expression in 17 PD blood samples versus healthy 11 controls belong to the GO Biological Function Protein Binding.
