# Supplementary material for: SRRM2, a Potential Blood Biomarker Revealing High Alternative Splicing in Parkinson's Disease
Source: PLoS One. 2010 Feb 8;5(2):e9104. doi: 10.1371/journal.pone.0009104 (PMC2817002; doi:10.1371/journal.pone.0009104)
Supplement: Table S5 — Characteristics of our PD cohort included in our Real-Time PCR analysis. M = Male, F = Female, A–R = Akinetic-Rigid dominant PD, Tremor = Tremor dominant PD, HY = Hoehn and Yahr Parkinson's disease stage, PMI = post-mortem interval. (0.05 MB DOC) [file pone.0009104.s007.doc]

| **Case** | **Age** | **Sex** | **Onset** | **PMI** | **Onset subtype** | **HY** |
| --- | --- | --- | --- | --- | --- | --- |
| PD1 | 66 | M | 61 | 5 | A-R | 4 |
| PD2 | 67 | M | 63 | 8.25 | A-R | 3 |
| PD3 | 85 | M | 60 | 5 | Tremor | 4 |
| PD4 | 82 | M | 79 | 5 | A-R | 3 |
| PD5 | 67 | M | 47 | 4 | Tremor | 4 |
| PD6 | 71 | M | 54 | 5.5 | Tremor | 5 |
| PD7 | 81 | F | 72 | 9.75 | A-R | 4 |
| PD8 | 74 | F | 60 | 9 | Tremor | 5 |
| PD9 | 85 | M | 79 | 2 | A-R | 3 |
| PD10 | 89 | M | 82 | 4 | Tremor | 4 |
| PD11 | 84 | M | 66 | 1 | A-R | 4 |
| PD12 | 73 | F | 56 | 23 | Tremor | 4 |
| PD13 | 72 | F | 45 | 15 | Tremor | 5 |
| PD14 | 66 | M | 48 | 5 | A-R | 4 |
| PD15 | 82 | M | 62 | 3 | Tremor | 5 |
| PD16 | 72 | M | 51 | 3 | Tremor | 4 |
| PD17 | 85 | F | 74 | 17 | A-R | 4 |
| PD18 | 83 | F | 75 | 5.5 | Tremor | 4 |
| PD19 | 85 | M | 71 | 5 | A-R | 4 |
| PD20 | 78 | M | 55 | 6 | Tremor | 4 |
| Mean | 77.3 | 14:06 | 63 | 7.05 |  | 4.05 |
| (SD) | 7.6 |  | 11.4 | 5.47 |  | 0.6 |

**Supplementary Table S5: Characteristics of our PD cohort included in our Real-Time PCR analysis.**

M= Male, F= Female, A-R= Akinetic-Rigid dominant PD, Tremor= Tremor dominant PD, HY=Hoehn and Yahr Parkinson’s disease stage, PMI= post-mortem interval.
